# Impact of partial occlusion of the face on multisensory emotion perception: Comparison of pre- and post-COVID-19 pandemic

**DOI:** 10.1371/journal.pone.0307631

**Published:** 2025-01-09

**Authors:** Misako Kawahara, Akihiro Tanaka

**Affiliations:** 1 Graduate School of Humanities and Sociology, The University of Tokyo, Tokyo, Japan; 2 Japan Society for the Promotion of Science, Tokyo, Japan; 3 Department of Psychology, Tokyo Woman’s Christian University, Tokyo, Japan; Kansai University, JAPAN

## Abstract

We perceive and understand others’ emotional states from multisensory information such as facial expressions and vocal cues. However, such cues are not always available or clear. Can partial loss of visual cues affect multisensory emotion perception? In addition, the COVID-19 pandemic has led to the widespread use of face masks, which can reduce some facial cues used in emotion perception. Thus, can frequent exposure to masked faces affect emotion perception? We conducted an emotion perception task using audio-visual stimuli that partially occluded the speaker’s face. Participants were simultaneously shown a face and voice that expressed either congruent or incongruent emotions and judged whether the person was happy or angry. The stimuli included videos in which the eyes or mouth were partially covered and where the whole face was visible. Our findings showed that, when facial cues were partially occluded, participants relied more on vocal cues for emotion recognition. Moreover, when the mouth was covered, participants relied less on vocal cues after the pandemic compared to before. These findings indicate that partial face masking and prolonged exposure to masked faces can affect multisensory emotion perception. In unimodal emotion perception from only facial cues, accuracy also improved after the pandemic compared to before for faces with the mouth occluded. Therefore, changes in the reliance on vocal cues in multisensory emotion perception during the pandemic period could be explained by improved facial emotion perception from the eye region.

## Introduction

The perception of linguistic and nonlinguistic information is required for successful communication among humans. Faces and voices are important sources of information for understanding others’ feelings. Furthermore, the processing of facial and vocal emotional cues results in emotion perception [1–3]. However, faces, sources of visual information, are not always evident in multisensory emotion recognition situations in our daily lives. Individuals may be partially unable to use facial cues when the expressor wears a sunglass or mask. Owing to the COVID-19 pandemic, people have been encouraged to wear face masks and cover their mouths and noses to minimize the risk of infection. Face masks cover approximately 65% of the face, which is crucial for facial emotion perception [4]. Hence, covering the eyes and mouth, which are important cues for detecting the affective state of others, causes a decline in the accuracy of facial emotion perception [5–7]. Recent studies demonstrated that partial occlusion of the face by facial masks was an obstacle to accurately reading emotions from facial expressions [4, 8, 9]. In the study by Carbon et al., for example, participants were shown pictures of six facial expressions (angry, disgust, fear, happy, neutral, and sad) with or without a face mask, and were asked to assess the depicted person’s emotional state from a list of six emotions. They found that the accuracy of facial expression recognition for anger, disgust, joy, and sadness was lower with masked faces than without masked faces. Moreover, studies examining the effects of transparent masks on facial expression recognition have reported decreased recognition accuracy for specific emotional expressions when the mouth is occluded [10]. In this study, participants were presented with pictures of female (Experiment 1) and male (Experiment 2) facial expressions (angry, disgust, fear, happy, neutral, sad, and surprise) with either a surgical mask, a transparent mask, or no mask and were asked to categorize the perceived facial emotion and rate the perceived intensity of emotion of the stimulus. The recognition accuracy for fearful and happy facial expressions was lower for faces with surgical masks than for those in other conditions. Furthermore, even when emotions were correctly classified, the perceived intensity of happiness was weaker for faces with surgical masks than for those in other conditions. In addition, Saito et al. [11] found that face masks hindered the recognition of specific emotions (i.e., fearful, sad, disgusted, and neutral) regardless of culture when comparing Japanese and American perceivers. These results demonstrated that partial occlusion of the face affected unimodal emotion perception from faces, although differences were observed depending on the type of emotion. However, in daily communication, individuals receive different emotional information, such as facial and vocal expressions, through each channel and integrate them into the emotion perception process. Therefore, a lack of facial information may also affect multisensory emotion perception and unimodal emotion perception from faces.

The effect of constraining visual information on multisensory emotion perception was examined by degrading visual information. Kokinous et al. [12] demonstrated that the integration of auditory and visual cues in emotion perception was influenced by the degradation of visual information, which they achieved by applying Gaussian filters to speakers’ faces. However, it remained unclear whether and how facial occlusion affected multisensory emotion perception. We examined how partial occlusion of the face modulated the weighting of faces and voices in multisensory emotion perception. We hypothesized that partial occlusion of the face would reduce visual cues and thus facilitate attention to vocal expressions when the eyes and mouth were covered, rather than when they were not (Hypothesis 1).

We examined the long-term effects of facial occlusion on multisensory emotion perception. Did the weighting of faces and voices in multisensory emotion perception change before and after the COVID-19 pandemic? During the COVID-19 pandemic, people dramatically increased their exposure to faces with obstructed lower halves in face-to-face communication. Barrick et al. [13] conducted experiments on people in the United States and provided evidence that exposure to masks altered the processing of facial emotions as it enhanced the reliance on visual facial cues, especially those around the eye. In East Asia, particularly in countries like Japan and China, adherence to social norms has driven the widespread adoption of mask-wearing [14, 15]. Notably, in Japan, the mask-wearing rate exceed 95% [16]. Japanese people may have a higher frequency of exposure to faces obstructed by masks. Based on the results of unimodal emotional perception from a face, the long-term effect of mask-wearing may also be observed in multisensory emotion perception. In other words, their weighting of facial and vocal cues may have changed before and after the COVID-19 pandemic. If mask exposure affected multisensory perception, there were two possible scenarios: A possibility was that the degree of the weighting of vocal cues in multisensory emotion perception increased after the pandemic (Hypothesis 2a). During mask exposure, the overall weighting of the auditory information may have increased to compensate for reduced visual cues. Another possibility was that the degree of weighting of vocal cues in multisensory emotion perception decreased after the pandemic (Hypothesis 2b). Ger et al. [17] investigated the effects of mask exposure time on the recognition accuracy of happy, sad, and angry facial expressions in 4–6-year-old children across two cultures with different levels of mask-wearing mandates (Brazil and Switzerland). They found that Brazilian children with longer exposure to masked faces demonstrated higher recognition accuracy for facial expressions than Swiss children. This result suggests that prolonged exposure to masked faces not only improves recognition accuracy for facial expressions with the lower face occluded but also enhances the overall recognition accuracy for facial expressions, including those with the upper face occluded or without any occlusion. Based on these findings, we hypothesize that in multisensory emotion perception, the accuracy of facial expressions as facial cues may have improved after exposure to masked faces compared to before, while the weighting of vocal cues may have decreased. Moreover, previous findings have suggested that mask exposure can promote reliance on information around the eye [13]. Building on these findings, we predicted that this reliance may enhance the accuracy of facial emotion perception from the upper half of the face, potentially reducing the weighting of vocal cues, particularly for speakers whose mouths are covered.

## Methods

### Participants

We conducted the same experiment with participants in June 2019 (prior to the COVID-19 pandemic, with the recruitment period from June 5, 2019, to June 21, 2019) and from November 2022 to January 2023 (post the COVID-19 pandemic, with the recruitment period from November 21, 2022, to January 25, 2023). The sample size was comparable between the pre- (*n* = 28, *Mage* = 20.4 years; *SD* = 0.6) and post-pandemic period (*n* = 29, *Mage* = 20.2 years; *SD* = 1.1). It should be noted that the data from the pre-pandemic period were originally obtained to examine cultural differences in multisensory emotion perception. Therefore, the sample size for the pre-pandemic experiment was initially determined using G*Power software to investigate cultural differences in vocal superiority in emotion recognition based on the results of Tanaka et al. [3], who utilized the same stimuli and tasks as those employed in this study. The results showed that a power of 80% could be achieved for a medium effect by using 28 participants in each cultural group. In this study, to compare results before and after the pandemic, data were collected from the same number of participants as in the pre-pandemic experiment for the post-pandemic data collection. All participants were native Japanese speakers and female students from the Tokyo Woman’s Christian University. They had normal hearing and normal or corrected-to-normal vision. This study was approved by the Research Ethics Board of the Tokyo Woman’s Christian University. All participants provided written informed consent before participating in the experiments. In this study, we retrospectively analyzed the data collected prior to the pandemic. Data from the experiment participants had already been de-identified, and consent forms were separated from the data. Only age and gender information were retained, ensuring that the authors could not identify individual participants. The data were accessed for research purposes from February 6, 2022, to June 10, 2023.

### Stimuli

We gathered the audiovisual stimuli from those used in our previous study [18]. We used emotional expressions of Japanese speakers to focus on emotion perception, with cultural in-group members for Japanese participants.

Stimuli were created from simultaneous audio and video recordings of Japanese speakers’ emotional utterances. In total, four short fragments with neutral linguistic meaning, such as “*Kore nani*?” (“*What is this*?”), “*Hai*, *moshimoshi*” (“*Hello*”), “*Sayonara*” (“*Good-bye*”), and “*Sounandesuka*?” (“*Is that so*?”) were uttered by two Japanese female speakers. Each fragment was spoken with a happy or angry emotional intonation, which are two emotions that are opposite in valence but similar in arousal level. Emotionally congruent and incongruent stimuli were created using audio and visual recordings. Unchanged recordings served as emotional congruent stimuli (i.e., angry faces with angry voices and happy faces with happy voices). For the emotional incongruent stimuli, happy and angry facial expressions were combined with angry and happy vocal expressions, respectively (i.e., angry faces with happy voices, happy faces with angry voices). The combination of facial and vocal expressions was conducted for each of the eight utterances (two speakers × four fragments), resulting in 32 bimodal stimuli (16 congruent and 16 incongruent). Although we primarily focused on responses for the incongruent stimuli to test our hypothesis, congruent stimuli were included and presented to prevent participants from noticing consistent incongruence and applying conscious strategies in the experiment.

**Fig 1:**
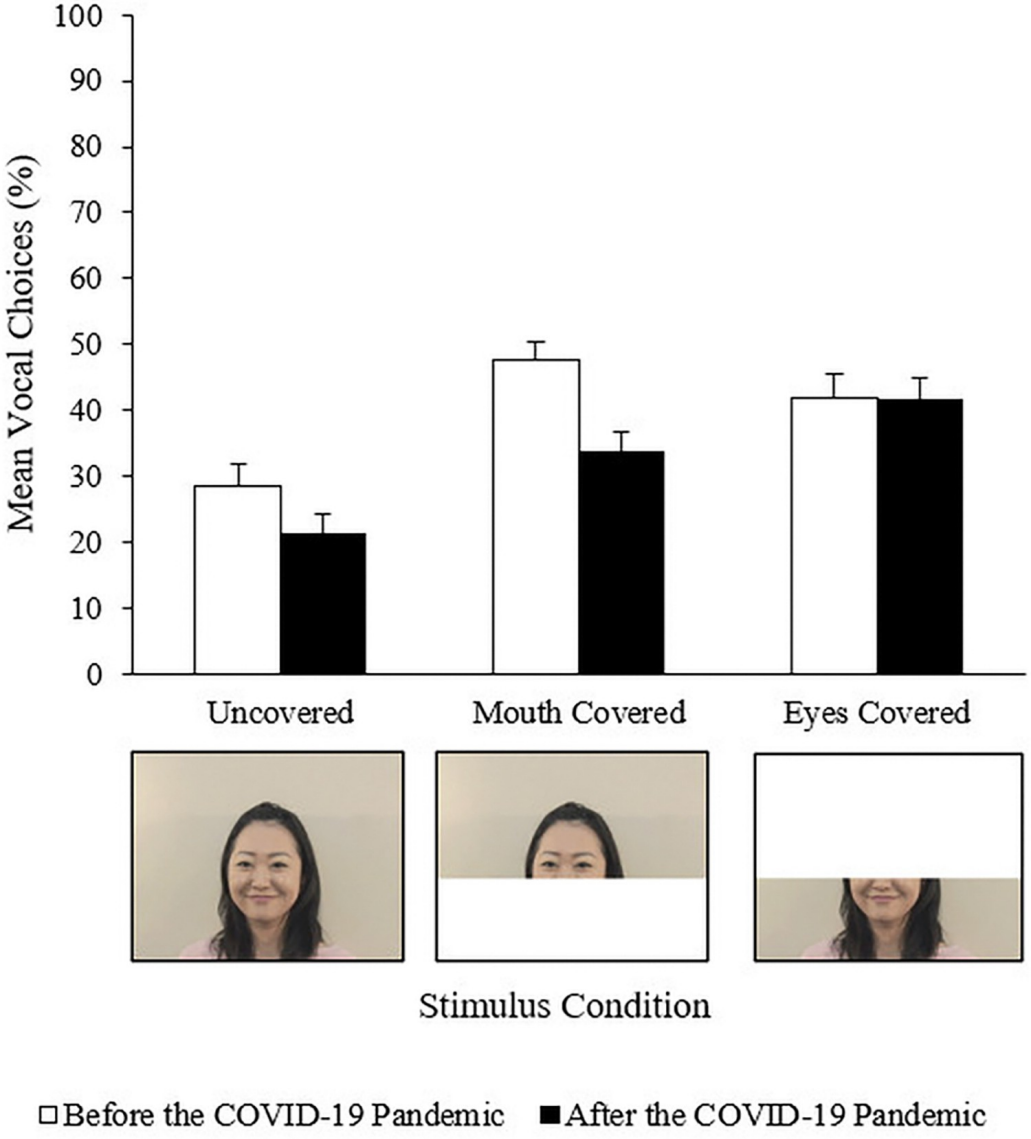
Example of experimental stimuli and the mean voice choice responses (VCs) in the affectively incongruent condition in each condition of period and occlusion of the face.

To examine how face visibility affected multisensory emotion perception, we manipulated the partial occlusion of the face in three conditions: uncovered, mouth covered, and eyes covered (Fig 1). A white square was digitally added to the original facial stimuli to obscure the area below and above the center of the dorsum of the nose for mouth-covered and eyes-covered stimuli, respectively, using Adobe Premiere Elements 2018 (Adobe Inc.). The original facial stimuli were used as uncovered stimuli. Hence, there were 96 bimodal stimuli (48 stimuli each [two speakers × four fragments × two combinations of facial and vocal expressions × three occlusions] were created with emotional congruence and incongruence). Unimodal stimuli were also created from audio or video recordings of speakers’ utterances, resulting in 64 unimodal stimuli (16 voice-only stimuli [two speakers × four fragments × two emotions] and 48 face-only stimuli [two speakers × four fragments × two emotions × three occlusions]). Therefore, 160 stimuli were presented once with no repetitions in the experiment.

### Procedure

Participants were assessed individually in quiet experimental rooms. The experiment consisted of three sessions that began with a multisensory session in which affective faces and voices were presented simultaneously (i.e., bimodal stimuli were presented). These were followed by two uni-sensory sessions in which only faces or voices (i.e., unimodal stimuli) were presented. In each trial, a fixation point was displayed, and a dynamic face (happiness or anger) and voice (happiness or anger) were presented simultaneously. The face was displayed on a PC monitor (Latitude3540, Dell) and the voice was presented binaurally via headphones (SENNHEISER HDA300) at a comfortable listening level, which was adjusted via headphone amplifiers (DAC-HA200, ONKYO). The order of trials was randomized. Participants were instructed to categorize the emotion of the speaker in the movies as happiness or anger. No instructions were provided regarding which modality the participants had to pay attention to. Therefore, there were no correct answers for the incongruent stimuli. For example, if a participant saw a happy face paired with an angry voice and their response was anger, we regarded their answer as a response that focused on the speaker’s voice (voice choice). Conversely, if a participant saw a happy face paired with an angry voice and their response was happy, we regarded their answer as a response that focused on the speaker’s face (face choice). Participants responded by pressing one of the two keys counterbalanced across them. All participants completed the main session after a practice session consisting of four trials.

### Transparency and openness

There were no data exclusions or manipulations not reported above, and all measures in the study are reported. We followed JARS [19]. All data are available at [https://osf.io/pqt7a/]. Data were analyzed using IBM SPSS Statistics 28. The study design and its analyses were not preregistered.

## Results and discussion

To examine how partial occlusion of the face influences the weighting of faces and voices in multisensory emotion perception and whether this weighting changes before and after the COVID-19 pandemic, we focused on the responses in affectively incongruent trials and calculated the percentage of voice choice responses (VCs) for incongruent expressions. The mean VC for each condition of facial occlusion and experimental time period for affectively incongruent stimuli is shown in Fig 1.

To examine the effect of face visibility and mask exposure during the COVID-19 pandemic on multisensory emotion perception, we performed Period (before and after the pandemic) × Facial occlusion (uncovered, mouth covered, and eyes covered) analysis of variances (ANOVAs) on the mean VC in affectively incongruent conditions. Results showed that the main effect of Facial occlusion (*F* (1.80, 99.12) = 44.25, *p* < .001, $\eta_{p}^{2}$ = .45) was significant. Bonferroni post-hoc tests showed that VCs were higher in the mouth-covered and eyes-covered conditions than in the uncovered condition (ps < .001). There was no significant difference between the mouth-covered and eyes-covered conditions (*p* = 1.00). These results supported Hypothesis 1. Our findings demonstrated that partial occlusion of the face affected multisensory emotion perception. Furthermore, we expanded on a previous study that reported that auditory cues were used more frequently when visual cues were degraded [12].

The main effect of Period was marginally significant (*F* (1, 55) = 3.22, *p* = .08, $\eta_{p}^{2}$ = .06), which indicated that VC was higher before the pandemic than after. Most importantly, the interaction between Period and Facial occlusion was significant (*F* (1.80, 99.12) = 6.00, *p* = .005, $\eta_{p}^{2}$ = .10). Simple main effect analyses demonstrated that the differences in VC between before and after the pandemic was observed in the mouth-covered condition (*F* (1, 55) = 10.47, *p* = .002, $\eta_{p}^{2}$ = .16); however, not in eyes-covered (*F* (1, 55) = 0.001, *p* = .98, $\eta_{p}^{2}$ < .001) and uncovered conditions (*F* (1, 55) = 2.72, *p* = .11, $\eta_{p}^{2}$ = .05). To examine the power of the interaction effect, we conducted a post-hoc power analysis using G*Power. The power (1-β) was equal to 0.99 when α = 0.05, and analysis parameters were set to a *F* test, which confirmed adequate power with the sample size. These results supported Hypothesis 2b and provided novel evidence of changes in multisensory emotion perception from face and voice during the COVID-19 pandemic. In addition, the simple main effect of Facial occlusion was significant both before (*F* (2, 54) = 23.28, *p* < .001, $\eta_{p}^{2}$ = .46) and after the pandemic (*F* (2, 56) = 27.06, *p* < .001, $\eta_{p}^{2}$ = .49). Bonferroni post-hoc tests showed that VCs were higher in the mouth-covered and eyes-covered conditions than in the uncovered condition, both before and after the pandemic (ps < .001). Interestingly, VCs were higher in the mouth-covered condition than in the eyes-covered condition before the pandemic (*p* < .05), whereas after the pandemic, VCs were lower in the mouth-covered condition than in the eyes-covered condition (*p* < .05).

One point to consider was whether the changes in VC during the pandemic period could be due to improved facial emotion recognition from the eyes. If this holds true, improved accuracy in emotion perception from uni-sensory facial cues should be observed only in the mouth covered condition, not uncovered and eyes covered conditions. To assess this possibility, we conducted a Period × Facial occlusion ANOVA on the mean accuracy in the uni-sensory face-only condition (Table 1).

**Table 1:** Mean accuracy in the affectively congruent, uni-sensory face-only, and uni-sensory voice-only condition in each condition of period (before and after the pandemic) and occlusion of the face (uncovered, mouth-covered, and eyes-covered).

	Multisensory session Affectively congruent		Uni-sensory session Face only		Uni-sensory session Voice only	
	Before	After	Before	After	Before	After
Uncovered	96.2 (1.0)	96.3 (1.4)	96.7 (0.8)	96.3 (0.9)	93.5 (1.1)	94.4 (1.2)
Mouth covered	93.1 (1.0)	95.0 (1.4)	87.3 (0.9)	90.7 (1.4)		
Eyes covered	97.3 (0.7)	95.7 (1.4)	96.4 (0.7)	95.5 (1.1)		

Values in parentheses represent the standard error.

Results showed that the main effect of Facial occlusion was significant (*F* (1.65, 90.88) = 47.60, *p* < .001, $\eta_{p}^{2}$ = .46). Bonferroni post-hoc tests showed that the accuracy was lower in the mouth-covered condition than in the eyes-covered and uncovered conditions (ps < .001). There was no significant difference between the eyes-covered and uncovered conditions (*p* = 1.00). The main effect of Period was not significant (*F* (1, 55) = 0.53, *p* = .47, $\eta_{p}^{2}$ = .01). Importantly, the interaction between Period and Facial occlusion was significant (*F* (1.65, 90.88) = 3.88, *p* = .03, $\eta_{p}^{2}$ = .001). Simple main effect analyses demonstrated that the differences in the accuracy in facial emotion perception before and after the pandemic was observed in the mouth-covered condition (*F* (1, 55) = 4.26, *p* = .04, $\eta_{p}^{2}$ = .07); however, not in the eyes-covered (*F* (1, 55) = 0.08, *p* = .78, $\eta_{p}^{2}$ = .001) and uncovered conditions (*F* (1, 55) = 0.50, *p* = .48, $\eta_{p}^{2}$ < .001). These results suggested that mask exposure could have enhanced the accuracy of facial emotion perception from the upper half of the face. Therefore, changes in VC during the pandemic period could be explained by improved facial emotion recognition from the eye area.

To examine whether the period (i.e., before and after the pandemic) and partial occlusion of faces affected the accuracy of recognizing emotions when the face and voice expressed the same emotion, we also performed Period × Facial occlusion ANOVAs on mean accuracy in affectively congruent conditions. The main effect of Facial occlusion was significant (*F* (2, 110) = 5.05, *p* = .008, $\eta_{p}^{2}$ = .08). Bonferroni post-hoc tests showed that the accuracy was lower for the mouth-covered condition than for the other two conditions (ps < .05). However, the main effect of Period (*F* (1, 55) = 0.01, *p* = .91, $\eta_{p}^{2}$ < .001) and interaction between Period and Facial occlusion (*F* (2, 110) = 2.24, *p* = .11, $\eta_{p}^{2}$ = .04) were not significant. In addition, there was no difference in accuracy before and after the pandemic, even in uni-sensory voice-only conditions (*t* (55) = -.52, *p* = .60).

We demonstrated that exposure to masked faces affected multisensory emotion perception and not only unimodal emotion perception from faces [12]. Differences in VCs before and after the pandemic suggested that emotions were perceived differently based on whether masks were worn, even if emotions were expressed similarly. In daily emotional expressions, faces and voices do not always match, and each can express different emotions [20]. Although facial expressions, especially smiles, can be used to conceal an individual’s actual emotional state, changes in one’s voice caused by emotions are difficult to hide [21]. Therefore, wearing masks may cause communication discrepancies. Negative emotions concealed by a smile may be perceived from voice less than before the pandemic.

However, several issues must be addressed in future studies. First, we achieved facial occlusion by adding a white square to the facial stimuli. We concluded that the change in vocal superiority in multisensory emotion perception before and after COVID-19 reflected exposure to masked faces. However, the impact of exposure to masked faces may include both the effect of frequently encountering faces with the lower half covered and the effect of changing stereotypes associated with the mask as an accessory. Considering that we used rectangles to occlude faces, we only examined the former and not the latter. Exposure to masked faces during the pandemic may have increased familiarity with the mask itself or with mask wearers, potentially changing stereotypes. For example, a study conducted during the COVID-19 global crisis revealed enhanced perceptions of trustworthiness and social desirability toward mask-wearing faces [22]. Furthermore, another study demonstrated that the proportion of respondents with negative associations toward mask-wearing, such as unhealthiness, decreased compared to the period before the pandemic [23]. Indeed, previous research has shown that accessories worn can affect emotion perceptions. Kret et al. [24] demonstrated that emotion perception from the eyes is influenced by contextual information, such as what is used to cover non-eye regions. They reported that, when the head and mouth of the same facial expression model were covered, the model was rated as less emotional when a niqab was used than when a black bar was used. This result suggests a contextual effect in emotion recognition, where women wearing a niqab may be perceived as more conservative and submissive and therefore evaluated as less emotionally expressive overall. Similarly, in this study, if a mask was used to cover the mouth instead of a rectangle, stereotypes associated with mask wearers may have served as contextual cues, potentially altering the perception of emotions.

Second, we manipulated only visual accessibility and did not control for auditory clarity. In actual face-to-face communication situations, such as when wearing a mask, the visible area of the face and clarity of voice are both reduced [25]. Further studies should examine how the weighting of facial and vocal cues was affected when both cues were diminished due to the use of masks.

Third, we only used happy and angry expressions. A more detailed analysis revealed that the improvement in the accuracy of emotion perception from only faces during the pandemic period was observed only for happy faces with the mouth occluded (see S2 Fig). No changes in accuracy were observed for happy faces with no occlusion or with the eyes occluded, or for angry faces. This lack of change in accuracy for these stimuli may be due to the ceiling effect, as there was little room for improvement in recognition accuracy. Furthermore, in multisensory emotion perception, changes in VC during the pandemic period were observed regardless of the combination of facial and vocal emotions (see S1 Fig). For example, although there was no improvement in the accuracy of facial emotion perception for angry faces with the mouth occluded, a reduction in VC was still observed for angry faces paired with happy voices during the pandemic period. These findings suggest that changes in multisensory emotion perception from faces and voices during the pandemic do not fully align with the improved accuracy of emotion perception from uni-sensory facial cues. Therefore, further investigation is necessary to determine whether similar patterns of facial emotion perception would be observed when using stimuli that increase the difficulty of facial perception or when using expressions such as fear, which involve characteristic cues across both the upper and lower parts of the face.

Fourth, we conducted the experiment only with Japanese participants. The results reported in this study (i.e., the change in vocal superiority in multisensory emotion perception before and after COVID-19) may not be limited to Japanese or other Asian populations but may also be applicable to other cultural groups. This is because the situation of being exposed to limited facial information (i.e., masked faces) was common across cultures during the COVID-19 pandemic. In fact, a previous study conducted with American participants reported that exposure to masks altered the processing of facial emotions as it enhanced the reliance on visual facial cues, especially those around the eye [13]. In addition, a study conducted with Hebrew speakers found that the accuracy of emotion recognition from the eye region improved after COVID-19 compared to before [26], which is consistent with the results of this study. However, Westerners tend to rely more on visual information than East Asians [3, 18], so the magnitude of change before and after COVID-19 may be smaller in Westerners than in Asians, which remains an issue for future investigation.

## Supporting information

S1 Fig The mean voice choice responses (VCs) in the affectively incongruent condition in each condition of period, occlusion of the face, and stimulus emotional combination. (DOCX)

S2 Fig The mean accuracy in uni-sensory face-only condition in each condition of period, occlusion of the face, and stimulus emotion. (DOCX)
