# Supplementary material for: Impact of partial occlusion of the face on multisensory emotion perception: Comparison of pre- and post-COVID-19 pandemic
Source: PLoS One. 2025 Jan 9;20(1):e0307631. doi: 10.1371/journal.pone.0307631 (PMC11717201; doi:10.1371/journal.pone.0307631)
Supplement: S1 Fig — (DOCX) [file pone.0307631.s001.docx]

**S1 Fig. The Mean Voice Choice Responses (VCs) in the Affectively Incongruent Condition in Each Condition of Period, Occlusion of the Face, and Stimulus Emotional Combination.**


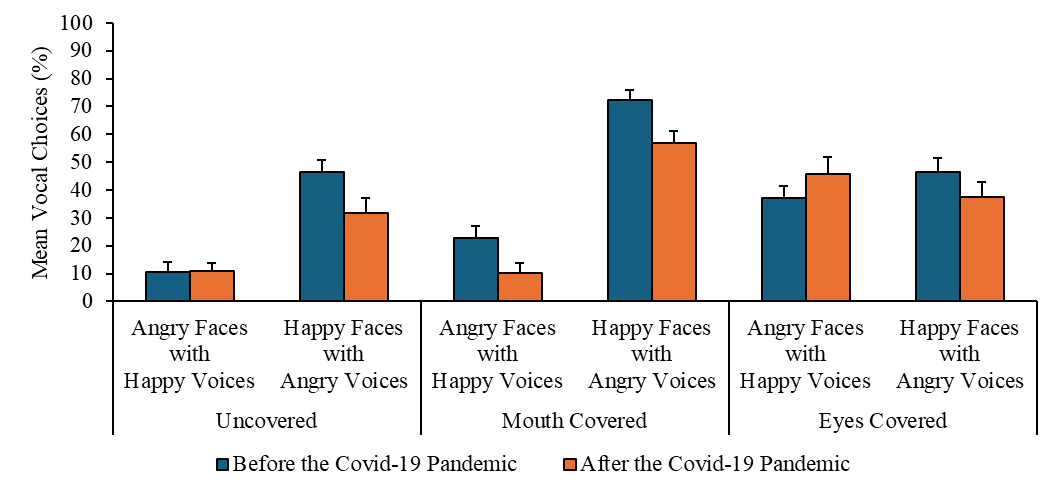


In our article, we did not consider the emotion of stimuli in our analysis of the effect of the visibility of the face and impact of the mask exposure during the COVID-19 pandemic in multisensory emotion perception. However, it has been shown that the mouth is important for the detection of happiness, while the eyes are more relevant for anger (e.g., Bombari et al., 2013; Calvo et al., 2014). Therefore, the effects of facial occlusion and exposure to masked faces (changes before and after the pandemic) may vary depending on the combination of facial and vocal emotions in the stimuli. To examine this possibility, we conducted a Period (before and after the pandemic) × Facial occlusion (uncovered, mouth covered, and eyes covered) × Emotion (angry face with happy voice and happy face with angry voice) analysis of variances (ANOVAs) on mean VC in the affectively incongruent condition (S1 Fig).

Similar to the two-way analysis (Period × Facial occlusion), results showed that the main effect of Facial occlusion was significant (*F* (2, 110) = 44.25, *p* < .001, $\eta_{p}^{2}$ = .45), and the main effect of Period was marginally significant (*F* (1, 55) = 3.22, *p* =.08, $\eta_{p}^{2}$ = .06). The interaction between Period and Facial occlusion was significant (*F* (1.80, 99.12) = 6.00, *p* =.005, $\eta_{p}^{2}$ = .10).

As for the results concerning the Emotion factor, the main effect of Emotion was also significant (*F* (1, 55) = 49.55, *p* < .001, $\eta_{p}^{2}$ = .47), which indicated that VC was higher for stimuli combining a happy face with an angry voice than for stimuli combining an angry face with a happy voice. These differences in VC observed across stimulus combinations are consistent with previous research findings (Tanaka et al., 2010; Kawahara et al., 2022). The interaction between Facial occlusion and Emotion was also significant (*F* (2, 110) = 60.55, *p* <.001, $\eta_{p}^{2}$ = .52). Simple main effect analyses showed that the differences in VC between combination of facial and vocal emotion was observed in the uncovered conditions (*F* (1, 55) = 55.92, *p* < .001, $\eta_{p}^{2}$ = *.*50) and mouth-covered condition (*F* (1, 55) = 177.62, *p* < .001, $\eta_{p}^{2}$ = *.*76); however, not in eyes-covered (*F* (1, 55) = 0.01, *p* = .92, $\eta_{p}^{2}$ = *.*0002). In addition, differences in VC across the conditions of facial occlusion were observed for both stimulus combinations. When participants were presented with stimuli combining an angry face and a happy voice (*F* (2, 110) = 58.43, *p* <.001, $\eta_{p}^{2}$ = .52), the VC was largest for the Eyes Covered condition, followed by the Mouth Covered condition, and smallest for the Uncovered condition. In contrast, when participants were presented with stimuli combining a happy face and an angry voice (*F* (2, 110) = 47.23, *p* <.001, $\eta_{p}^{2}$ = .46), the VC was larger for the Mouth Covered condition compared to both the Uncovered and Eyes Covered conditions. These results suggest that VC increases due to partial facial occlusion for both combinations of facial and vocal emotions. In particular, the increase in VC is pronounced for stimuli combining a happy face with an angry voice when the mouth, which is crucial for recognizing a smile, was occluded. Importantly, however, the interaction between Period and Emotion (*F* (1, 55) = 2.58, *p* =.11, $\eta_{p}^{2}$ = .05) and the three-way interaction (*F* (2, 110) = 1.58, *p* =.21, $\eta_{p}^{2}$ = .03) was not significant. Therefore, the effects of exposure to masked faces (changes before and after the pandemic) did not vary by the combination of facial and vocal emotions in the stimuli.
