# Supplementary material for: Impact of partial occlusion of the face on multisensory emotion perception: Comparison of pre- and post-COVID-19 pandemic
Source: PLoS One. 2025 Jan 9;20(1):e0307631. doi: 10.1371/journal.pone.0307631 (PMC11717201; doi:10.1371/journal.pone.0307631)
Supplement: S2 Fig — (DOCX) [file pone.0307631.s002.docx]

**
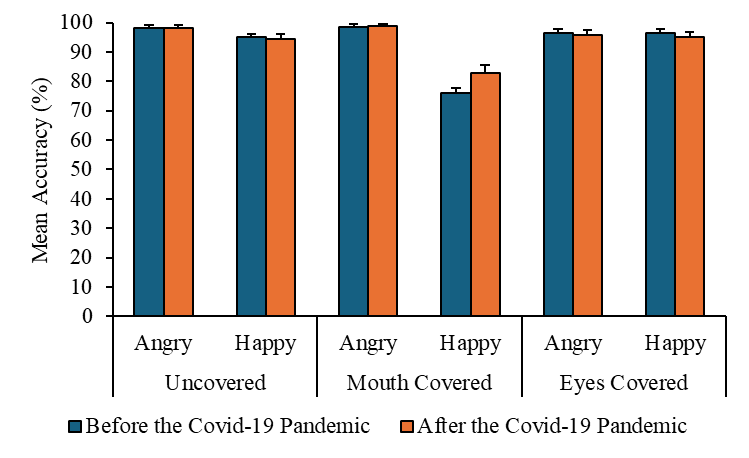
S2 Fig. The Mean Accuracy in Uni-sensory Face Only Condition in Each Condition of Period, Occlusion of the Face, and Stimulus Emotion.**

To examine whether the effects of facial occlusion and exposure to masked faces on facial emotion perception accuracy vary depending on the facial emotion (angry or happy), we conducted a Period (before and after the pandemic) × Facial occlusion (uncovered, mouth covered, and eyes covered) × Emotion (angry and happy face) analysis of variances (ANOVAs) on mean accuracy in uni-sensory face only condition (S2 Fig).

Similar to the two-way analysis (Period × Facial occlusion), results showed that the main effect of Facial occlusion was significant (*F* (2, 110) = 47.60, *p* < .001, $\eta_{p}^{2}$ = .46), while the main effect of Period was not significant (*F* (1, 55) = 0.53, *p* =.47, $\eta_{p}^{2}$ = .001). The interaction between Period and Facial occlusion was significant (*F* (2, 110) = 3.88, *p* =.02, $\eta_{p}^{2}$ = .07).

As for the results concerning the Emotion factor, the main effect of Emotion was also significant (*F* (1, 55) = 37.26, *p* < .001, $\eta_{p}^{2}$ = .40), which indicated that the accuracy was higher for angry face than for happy face. The interaction between Facial occlusion and Emotion was also significant (*F* (2, 110) = 74.05, *p* <.001, $\eta_{p}^{2}$ = .57). Simple main effect analyses showed that the accuracy was higher for angry faces than for the happy faces in the uncovered conditions (*F* (1, 55) = 7.82, *p* = .007, $\eta_{p}^{2}$ = *.*12) and mouth-covered condition (*F* (1, 55) = 177.63, *p* < .001, $\eta_{p}^{2}$ = *.*68); however, not in eyes-covered (*F* (1, 55) = 0.02, *p* = .90, $\eta_{p}^{2}$ = *.*0003). In addition, differences in the accuracy across the conditions of facial occlusion were observed for both angry (*F* (2, 110) = 3.24, *p* =.04, $\eta_{p}^{2}$ = .06) and happy faces (*F* (2, 110) = 102.85, *p* <.001, $\eta_{p}^{2}$ = .65). Bonferroni post-hoc tests showed that no significant differences among the facial occlusion conditions in accuracy for angry stimuli. However, the accuracy for happy faces was lower in the mouth-covered condition compared to both the uncovered and eyes-covered conditions. These results suggest that the accuracy of facial emotion perception for happy faces decreases when the mouth, which is crucial for recognizing a smile, is occluded.

Although the interaction between Period and Emotion was not significant (*F* (1, 55) = 0.56, *p* =.46, $\eta_{p}^{2}$ = .01), the three-way interaction was significant (*F* (2, 110) = 3.24, *p* =.04, $\eta_{p}^{2}$ = .06). To examine whether differences in facial emotion perception accuracy based on the occluded facial region and period vary depending on the stimulus emotions, we conducted a simple interaction analysis involving Period × Facial occlusion on the mean accuracy for each condition of facial emotion (angry and happy). The results showed that the simple interaction between Period and Facial occlusion was significant for happy faces (*F* (2, 110) = 6.13, *p* = .003, $\eta_{p}^{2}$ = *.*10); however, not for angry faces (*F* (2, 110) = 0.09, *p* = .92, $\eta_{p}^{2}$ = *.*002). The results of the simple-simple main effect analysis showed that, the accuracy for happy faces was lower in the mouth-covered condition compared to the uncovered and eyes-covered conditions both before (*F* (2, 54) = 91.08, *p* < .001, $\eta_{p}^{2}$ = *.*77) and after the pandemic (*F* (2, 54) = 26.32, *p* < .001, $\eta_{p}^{2}$ = *.*48). However, the accuracy in the mouth-covered condition was higher after the pandemic than before (*F* (1, 55) = 4.49, *p* = .04, $\eta_{p}^{2}$ = *.*08), and such period-related differences were not observed in the uncovered (*F* (1, 55) = 0.11, *p* = .74, $\eta_{p}^{2}$ = *.*002) and eyes-covered conditions (*F* (1, 55) = 0.33, *p* = .57, $\eta_{p}^{2}$ = *.*006). Therefore, the improvement in facial emotion recognition accuracy due to exposure to masked faces was observed only for happy faces, not angry faces. The lower accuracy for happy faces in the mouth-covered condition can likely be explained by the fact that the mouth, which is crucial for recognizing happiness, was occluded. The finding that accuracy for happy faces with the mouth covered was higher after the pandemic than before suggests the influence of exposure to masked faces. In contrast, for angry faces, no differences were observed based on the occlusion condition or the period, likely due to a ceiling effect.
